# Trends in breast cancer screening rates among Korean women: results from the Korean National Cancer Screening Survey, 2005-2020

**DOI:** 10.4178/epih.e2022111

**Published:** 2022-11-24

**Authors:** Soo Yeon Song, Yun Yeong Lee, Hye Young Shin, Bomi Park, Mina Suh, Kui Son Choi, Jae Kwan Jun

**Affiliations:** 1 National Cancer Control Institute, National Cancer Center, Goyang, Korea; 2 Graduate School of Cancer Science and Policy, National Cancer Center, Goyang, Korea; 3 Department of Nursing, KC University, Seoul, Korea; 4 Department of Preventive Medicine, Chung-Ang University College of Medicine, Seoul, Korea

**Keywords:** Breast neoplasms, Early detection of cancer, Health care surveys, Mammography, Mass screening

## Abstract

**OBJECTIVES:**

Since 2002, the Korean government has provided breast cancer screening as part of the National Cancer Screening Program. This study reported trends in the screening rate among Korean women from 2005 to 2020, including organized and opportunistic screening for breast cancer.

**METHODS:**

Data from the Korean National Cancer Screening Survey, an annual cross-sectional nationwide survey, were collected using a structured questionnaire between 2005 and 2020. The study population included 23,702 women aged 40-74 years with no history of cancer. We estimated the screening rate based on the current recommendation of biennial mammographic screening for breast cancer. In addition, a joinpoint trend analysis was performed for breast cancer screening rates among various subgroups.

**RESULTS:**

In 2020, the breast cancer screening rate was 63.5%, reflecting an annual increase of 7.72% (95% confidence interval 5.53 to 9.95) between 2005 and 2012, followed by non-significant trends thereafter. In particular, a significant decrease in the breast cancer screening rate was observed in the subgroups aged 50-59 years old, with 12-15 years of education, and living in rural areas.

**CONCLUSIONS:**

Although there has been substantial improvement in breast cancer screening rates in Korean women, the trend has flattened in recent years. Therefore, continual efforts are required to identify subgroups with unmet needs and solve barriers to the uptake of breast cancer screening.

## INTRODUCTION

Breast cancer is a major cause of cancer-related mortality and disease burden among Korean women. In 2018, 23,547 new breast cancer cases were diagnosed, and the age-standardized rate for women breast cancer mortality increased from 4.2 per 100,000 in 1999 to 5.3 per 100,000 in 2018. Since 1999, the incidence of breast cancer in Korean women has increased from 11.0 per 100,000 in 1999 to 29.1 per 100,000 in 2018, although the increase in the annual percent change (APC) has slowed since 2007 [1]. From 2011 to 2015, the total annual costs for breast cancer patients increased by 34%, from 780.1 million dollars to 1,044.3 million dollars [2].

Breast cancer screening has been proven effective in reducing breast cancer mortality [3]. In Korea, both organized and opportunistic breast cancer screening programs have been developed. Since 2002, the National Cancer Screening Program (NCSP) in Korea has provided free or low-cost screening for breast cancer [4]. Individuals enrolled in the Medical Aid program and National Health Insurance (NHI) beneficiaries can receive services free of charge if their premium scale is less than 50%. In contrast, NHI beneficiaries in the top 50% of the premium scale pay 10% of the screening cost. Conversely, individuals who use opportunistic cancer screening programs pay all procedural expenses [2]. Although the Korean government provides organized cancer screenings, many Koreans still undergo opportunistic screenings despite the out-of-pocket costs [5].

Under the National Cancer Screening Guideline for breast cancer screening revised in 2015, the NCSP provides mammograms every 2 years for women aged ≥ 40 years [6,7]. Conversely, opportunistic breast cancer screening and the frequency and screening method used depend on individuals’ choices and their doctors [4]. In this study, based on the national recommendations for breast cancer screening, we report the overall breast cancer screening rate from both organized and opportunistic screening in Korea. We also analyzed the changing trends in screening rates by various demographic and socioeconomic factors that might be associated with breast cancer screening.

## MATERIALS AND METHODS

### Data source and study population

Since 2004, the National Cancer Center has conducted the Korean National Cancer Screening Survey (KNCSS), an annual national survey on screening behaviors and related factors for stomach, colorectal, liver, breast, and cervical cancers. The eligible population for the KNCSS comprised cancer-free men aged 40-74 years and cancer-free women aged 30-74 years, while cancer-free women aged 20-29 years have also been surveyed since 2015. The subjects were sampled based on the Resident Registration Population data using a stratified, multistage, random sampling procedure according to the residential area, gender, and age, to ensure that the survey participants were a representative population sample. The Korea National Statistical Office publishes the Resident Registration Population data annually after data are gathered from residents of the registration population on December 31 each year. Details of the sampling method have been described fully elsewhere [8]. We used the KNCSS data collected through face-to-face interviews, except for 2004, when data were collected via computer-assisted telephone interviews. Specialized research institutions recruited participants through in-person contact. At least 3 contact attempts were made for each household, and 1 person was selected from each household. All participants were fully informed and consented to participate in the survey for public purposes. According to the protocol of the NCSP, women aged > 40 years are eligible to undergo breast cancer screening. Therefore, this study included cancer-free women aged 40-74 who had participated in the KNCSS between 2005 and 2020. A total of 23,702 women were included in the final analysis (Supplementary Material 1).

### Variable definition

The KNCSS survey collected information on the history of screening for 5 types of cancer (stomach, liver, colorectal, breast, and cervical cancer) and socio-demographic characteristics using a structured questionnaire. The major questions about the participant’s cancer screening experiences are provided in Supplementary Material 2. The questions were as follows: “Have you ever undergone (cancer type) screening?”, “Which screening method was used?”, “When did you last undergo (cancer type) screening with this method?”, and “How did you pay for the screening?” Based on the screening payment method, the screened respondents were classified according to whether they underwent organized or opportunistic screening. If the payment method was any of the following, the respondent was considered to have undergone organized screening: “National Health Insurance/National Health Insurance Corporation (partially self-paid),” or “Public health center/government (total free of charge).” Respondents with other payment methods were considered to have undergone opportunistic screening. Socio-demographic factors such as age, monthly household income level, education level, and location of residence were considered. The age groups were 40-49 years, 50-59 years, 60-69 years, and 70-74 years. The monthly household income level was classified as low, middle, or high, according to the annual tertile. The educational level was divided into 3 groups according to the number of years of education received: ≤ 11 years (less than high school graduate), 12-15 years (high school graduate to less than 4-year college graduate), and ≥ 16 years (4-year college graduate or more). Finally, residential areas were classified as metropolitan, urban, or rural.

### Statistical analysis

To analyze the breast screening rates according to the recommendations, we estimated the proportion of respondents who reported having had a mammographic screening within the past 2 years, according to the NCSP Breast Cancer Screening Protocol (Table 1). To yield unbiased estimates, we calculated the breast cancer screening rates using survey sampling weights. Changes in breast cancer screening rates were estimated using joinpoint regression [9]. Both the APC and average annual percent change (AAPC) were calculated with 95% confidence intervals (CIs). The results were summarized as APCs using a linear model of each screening rate’s raw values. The AAPC is based on the weighted average of the APC, calculated across the joinpoints. Breast cancer screening trends were also calculated across the selected demographic and geographic subgroups. We adopted a maximum of 2 possible joinpoints for all analyses and selected the model that best explained the data trends [10]. Based on these results, we characterized breast cancer screening trends. The trend was described as stable if there was a change of 0.5% or less per year (-0.5%≤ APC≤ 0.5%) and if the APC was not statistically significant. If the APC changed by ≥ 0.5% per year (APC < -0.5% or APC> 0.5%) and the APC was not statistically significant, it was described as a non-significant change. A change with a statistically significant APC> 0 was described as increasing. A change to a statistically significant APC< 0 was described as decreasing [11]. Statistical analysis was performed using SAS version 9.4 (SAS Institute Inc., Cary, NC, USA) and Joinpoint version 4.8.0.1 (National Cancer Institute, Bethesda, MD, USA).

### Ethics statement

This study was approved by the Institutional Review Board of National Cancer Center (IRB approval No. NCC2019-0233). All participants were fully informed and consented to participate in the survey for public purposes.

## RESULTS

**Figure 1.**
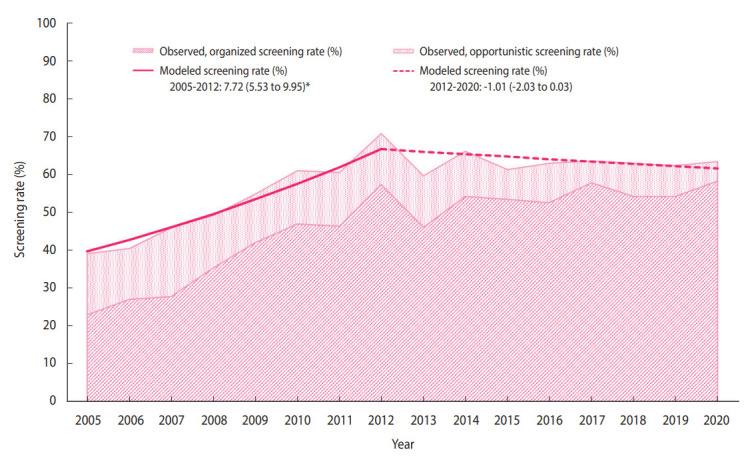
Trends in breast cancer screening rates according to the recommendations, 2005-2020. The area chart denotes the observed organized and opportunistic screening rates. The solid line denotes a significantly increasing trend; the densely dotted line denotes a non-significant change. Values are presented as annual percent change (95% confidence interval). ^*^p-value for the trend in annual percent change <0.05

The distribution of the sociodemographic characteristics of the annual survey respondents is provided in Supplementary Material 3. The breast cancer screening rate initially increased from 38.4% in 2005 to 71.0% in 2012 (APC, 7.72%), followed by non-significant changes (Figure 1). In 2020, the breast cancer screening rate was 63.5% (Table 2), of which the proportions of the women who underwent organized and opportunistic screening were 58.3% and 5.2%, respectively (Supplementary Materials 4-6). Although the organized screening rate has slowed since 2010, a general increasing trend was observed (APC, 16.8% from 2005 to 2010; 1.7% from 2010 to 2020). Contrastingly, the opportunistic screening rate showed a decreasing trend (APC, -6.0% from 2005 to 2020). In all subgroups, the organized screening rates increased significantly, except in the age group of 70-74 years and the rural population.

**Figure 2.**
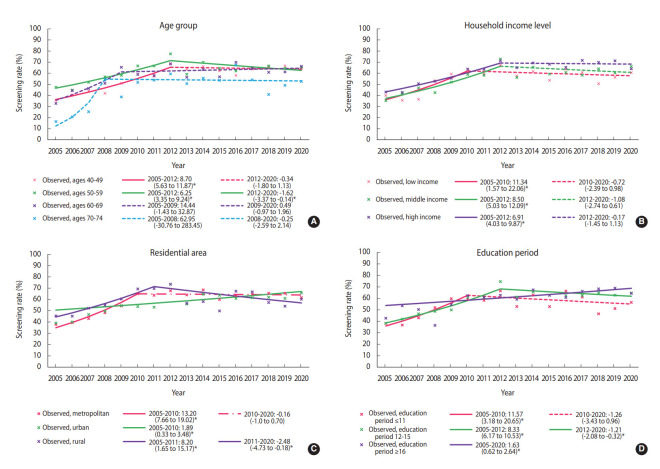
Trends in breast cancer screening rates in accordance with recommendations by subgroup, 2005-2020. The marker (×) denotes the observed screening rate. A solid line denotes a significantly increasing or decreasing trend; a densely dotted line denotes a non-significant change; a dash-dotted line denotes a stable trend. (A) The pink, green, purple, and blue lines denote the trends in women aged 40-49 years, 50-59 years, 60-69 years, and 70-74 years, respectively. (B) The pink, green, and purple lines denote the trends in women of low, middle, and high-income levels, respectively. (C) The pink, green, and purple lines denote the trends in women living in metropolitan, urban, and rural areas, respectively. (D) The pink, green, and purple lines denote the trends in women with 11 years or less, 12-15 years, and 16 years or more of education, respectively. Values are presented as annual percent change (95% confidence interval). ^*^p-value for the trend in annual percent change <0.05.

Until 2012, the screening rate for breast cancer significantly increased in almost all subgroups, followed by a plateau. However, since 2012, the breast cancer screening rate has decreased in women aged 50-59 years, having 12-15 years of education, and living in rural areas. In women aged 50-59 years, breast cancer screening rates showed a statistically non-significant increase until 2012, and then significantly decreased (APC, -1.62%; 95% CI, -3.07 to -0.14) (Figure 2A). In the low-income group, breast cancer screening rates increased at an annual average of 11.34% between 2005 and 2010, but there was no significant change in the rates afterward (APC, -1.08%; 95% CI, -2.74 to 0.61) (Figure 2B). The group with 12-15 years of education showed an increasing breast cancer screening trend until 2012, but the trend then decreased (APC, 8.33% from 2005 to 2012; -1.21% from 2012 to 2020) (Figure 2C). The rates in rural areas showed a consistently significant increase until 2011 and then showed a significant decreasing trend with fluctuations (APC, 8.20% from 2005 to 2011; -2.48% from 2011 to 2020) (Figure 2D).

## DISCUSSION

It is necessary to screen repeatedly at an optimal interval to detect breast cancer in its early stages. Therefore, cancer screening should be performed according to the recommended protocol. According to the recommendations, this statistical study reports time trends in breast cancer screening, considering both organized and opportunistic screening over the past 16 years. Furthermore, we analyzed the breast cancer screening trend in socio-demographic subgroups to determine whether specific subgroups had different trends. Between 2005 and 2020, the national breast cancer screening rate has increased considerably. It has been consistently maintained above 60% since 2010, increasing to 63.5% in 2020.

In response to reduced requirements for the designation of screening facilities in the NCSP for breast cancer in 2008, the number of screening facilities increased [12]. This increase in screening facilities would have contributed to the marked increase in NCSP participation for breast cancer screening, which was approximately 62% in 2017 [13]. Although the screening protocols are diverse, in recent years, the rates of breast cancer screening in accordance with the recommendations have flattened in the United States and England [14,15]. Korea’s breast cancer screening rates were slightly lower than those of the United States (77.2% in 2000, 74.2% in 2015) and England (77.2% in 2011, 75.0% in 2019) [14,15], and were higher than those in Japan (23.3% in 2004 and 42.3% in 2016) and the average rates of Organization for Economic Cooperation and Development member countries (55.3% in 2005 and 60.8% in 2015) [16].

We selected the joinpoint regression model that best described each data set in the subgroup analysis according to socioeconomic characteristics. Each group showed different changes in the breast cancer screening rate. The recent significant declining trend in the estimated screening rate among women aged 50-59 years might have been due to a temporary drop in the screening rate due to coronavirus disease 2019 (COVID-19) pandemic in 2020 [17]. Further investigations are needed to minimize the impact of COVID-19 for a more accurate estimation of trends in the breast cancer screening rate.

Income and education levels are significantly associated with participation in breast cancer screening [18]. In our results, as in previous studies [8,19], the breast cancer screening rate was relatively high in high-income households, and the recent trend in the breast cancer screening rates showed non-significant changes or remained stable for all household income levels. The fact that the screening rate did not decrease in low-income households may be due to the positive effect of free national cancer screening. According to the KNCSS data, the rate of free-of-charge breast screening in accordance with the recommendations, also called “organized screening,” showed a significant increase between 2005 and 2020. In contrast, opportunistic screening showed a significant decrease (Supplementary Material 6). This difference between the organized and opportunistic screening rates may be due to the relatively reduced demand for opportunistic screening, especially in low-income households, as systematic screening is well established.

Our results show that breast cancer screening increased significantly in urban areas between 2005 and 2020, but declined significantly after 2011 in rural areas, while there was no significant change in metropolitan areas after a significant increase between 2005 and 2010. Regional differences in breast cancer screening rates may be based on access to screening institutions. In Korea, mammography equipment is concentrated in large cities [12,20]. Therefore, it is important to resolve this distributional inequity in mammography facilities. The gradient of breast cancer screening rates between regions results from both differences in socioeconomic status and accessibility, so neither factor is a simple proxy for the other [21]. Therefore, these results should be interpreted with caution. Nevertheless, it has been reported that there is a gap in the rate of breast cancer screening in countries with and without an organized national screening program like that of Korea [22,23].

The KNCSS has reported nationwide breast cancer screening rates for over 16 years. However, there were some limitations to our study. First, there may have been recall bias as survey data were used. However, despite this methodological limitation of the survey, the KNCSS is used as the source for calculating representative indicators related to cancer screening in Korea [24]. Second, there is a possibility of sampling error. We adopted stratified multistage, random sampling according to the residential area, gender, and age. In addition, this nationwide, annual, population-based survey has been conducted since 2004. Therefore, the sampling error would gradually be minimized as the sample size increased and the data sufficiently represented the population. Third, the breast cancer screening rates were presented considering both organized and opportunistic screening, but we did not analyze trends in these 2 screening rates separately according to socioeconomic status because we performed this analysis to determine how well the breast cancer screening recommendations, which recommends biennial mammography for women aged 40 and older, was implemented, regardless of the payment method.

This study provided more comprehensive results by including an individual’s opportunistic screening experience besides their organized programmatic screening experiences. We report breast cancer screening rates between 2005 and 2020. Despite the continuous expansion of the breast cancer screening program, the mortality and medical cost burden of breast cancer is expected to increase in the future due to an increase in the absolute number of breast cancer patients (5,839 cases in 1999 vs. 23,723 cases in 2018) [1,25]. Additionally, decreasing trends in the breast cancer screening rate were observed for women aged 50-59 years, having 12-15 years of education, and living in rural areas. Further studies are needed to identify the groups more likely to be excluded from screening benefits and the causes of disparities in screening, including socio-demographic changes and gradients in breast cancer screening rate trends.

## Figures and Tables

**Table 1.** The Korean Guideline for Breast Cancer Screening and the protocol issued by the National Cancer Screening Program (NCSP) for breast cancer

Year of issue	Korean Guideline for Breast Cancer Screening		Protocol of the NCSP for breast cancer	
	2001 [6]	2015 [7]	2002	2016
Target population	Women aged ≥30 yr	Women aged 40-69 yr	Women aged ≥40 yr	Women aged ≥40 yr
Test	BSE (aged ≥30 yr)	MMG	MMG (CBE recommended only)	MMG
	CBE (aged ≥35 yr)			
	MMG (aged ≥40 yr)			
Interval	BSE: every month	2 yr	2 yr	2 yr
	MMG, CBE: 1-2 yr			
Additional study for confirmation			Fine-needle aspiration and biopsy	-^1^

BSE, breast self-examination; CBE, clinical breast examination; MMG, mammography.

1 Deleted from the NCSP for breast cancer since 2008.

**Table 2.** Breast cancer screening rates (%) in Korea, 2005-2020

Variables		Survey year																AAPC (95% CI)
		2005	2006	2007	2008	2009	2010	2011	2012	2013	2014	2015	2016	2017	2018	2019	2020	
Total		38.4	40.6	45.8	49.3	55.2	61.1	60.4	71.0	59.7	66.0	61.2	62.9	63.6	63.1	62.3	63.5	2.97 (1.96, 3.99)^*^
Age (yr)^1^																		
	40-49	35.8	40.6	44.1	42.3	51.0	59.4	57.4	69.0	63.8	66.1	62.3	58.2	63.9	65.9	66.8	64.2	3.78 (2.36, 5.23)^*^
	50-59	48.7	45.0	52.0	56.9	58.2	66.7	66.7	77.6	59.2	70.0	64.6	62.2	64.9	66.7	64.7	65.6	1.98 (0.61, 3.37)^*^
	60-69	34.1	44.3	46.6	52.0	65.6	59.1	58.1	68.5	56.5	63.5	56.9	70.0	64.6	61.0	61.3	66.4	4.03 (0.28, 7.92)^*^
	70-74	16.7	20.9	25.5	54.8	38.3	52.1	54.0	59.6	50.8	55.7	53.8	67.5	54.2	41.0	49.3	52.8	10.04 (-5.60, 28.27)
Education (yr)																		
	≤11	38.6	37.3	43.6	52.4	60.2	59.9	58.7	66.9	53.4	63.4	53.3	66.7	61.6	47.1	51.6	57.0	2.84 (0.13, 5.63)^*^
	12-15	38.9	42.5	47.1	49.2	50.5	61.2	61.0	75.0	61.6	66.1	63.2	62.7	63.0	65.2	63.2	65.1	3.13 (2.17, 4.11)^*^
	≥16	43.3	54.0	50.8	37.0	55.1	62.8	61.3	63.2	59.6	67.8	63.1	61.2	66.6	68.7	69.3	64.8	1.63 (0.62, 2.64)^*^
Monthly household income^2,3^																		
	Low	40.1	35.8	36.7	53.4	59.4	60.5	59.4	67.5	57.8	61.7	53.8	63.8	60.8	50.7	56.5	60.6	3.15 (0.19, 6.19)^*^
	Middle	35.5	42.2	47.0	42.7	52.1	59.1	58.3	73.0	56.4	65.6	59.4	60.8	58.4	64.1	60.5	66.3	3.28 (1.67, 4.92)^*^
	High	43.3	42.9	50.7	52.9	55.7	64.0	63.8	71.3	65.2	69.4	68.3	65.4	71.7	70.1	71.3	64.1	3.08 (1.75, 4.41)^*^
Residential area																		
	Metropolitan	37.9	39.5	43.1	48.3	54.3	65.1	63.8	67.8	64.1	68.6	60.0	63.6	64.9	65.6	65.6	61.8	4.11 (2.49, 5.76)^*^
	Urban	39.2	40.1	46.8	49.7	54.7	53.8	53.2	73.5	56.0	64.7	63.8	61.0	61.6	62.0	61.0	66.0	1.89 (0.33, 3.48)^*^
	Rural	42.5	45.3	52.3	55.0	60.5	69.4	69.8	73.6	56.7	58.2	49.9	67.4	66.9	57.4	54.1	60.3	1.66 (0.90, 4.28)

AAPC, average annual percent change; CI, confidence interval.

1 Respondents were restricted to women aged 40-74 years who had undergone mammography screening within a period of 2 years.

2 Low-income, middle-income, and high-income groups were classified according to each year’s tertile of household income.

3 Some columns do not sum to 100% because of missing data.

* p<0.05.

## SUPPLEMENTARY MATERIALS

Supplementary Material 1. Selection of analysis subjects flowchart by the year 2005–2020.

Supplementary Material 2. Korean National Cancer Screening Survey Questionnaire, 2020.

Supplementary Material 3. Distributions (%) of the sociodemographic characteristics of the respondents according to the Korean National Cancer Screening Survey (KNCSS), 2005–2020

Supplementary Material 4. Number of respondents underwent organized screening and the organized screening rates (%) by the sociodemographic characteristics according to the Korean National Cancer Screening Survey (KNCSS), 2005–2020

Supplementary Material 5. Number of respondents underwent opportunistic screening and the opportunistic screening rates (%) by the sociodemographic characteristics according to the Korean National Cancer Screening Survey (KNCSS), 2005–2020

Supplementary Material 6. Trends in breast cancer screening rates with recommendation by the payment method, 2005–2020. The marker (×) denotes an observed screening rate. The solid line denotes a significantly increasing or decreasing trend; the densely dotted line denotes a non-significant change. ^*^ p-value for the trend in annual percent changes (APCs) <0.05. (A) The pink and green lines denote the trends in women who underwent organized screening and women who underwent opportunistic screening, respectively.
