# Supplementary material for: Trends in breast cancer screening rates among Korean women: results from the Korean National Cancer Screening Survey, 2005-2020
Source: Epidemiol Health. 2022 Nov 24;44:e2022111. doi: 10.4178/epih.e2022111 (PMC10396513; doi:10.4178/epih.e2022111)
Supplement: Supplementary Material 1. — Selection of analysis subjects flowchart by the year 2005–2020. [file epih-44-e2022111-Supplementary-1.pptx]

## Slide 1
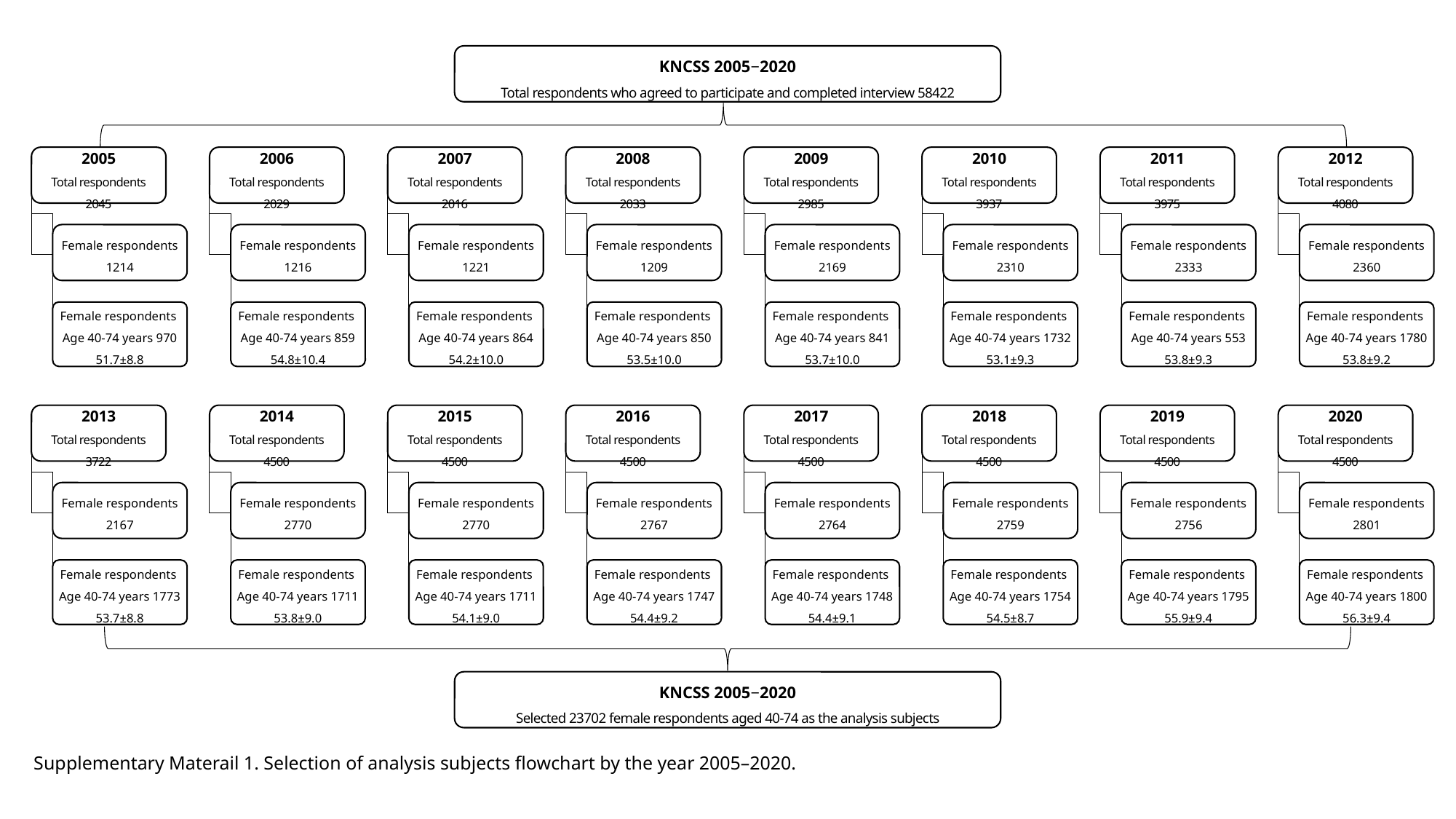

KNCSS 2005−2020
Total respondents who agreed to participate and completed interview 58422
2005
Total respondents 2045
Female respondents 1214
Female respondents
Age 40-74 years 970
51.7±8.8
2006
Total respondents 2029
Female respondents 1216
Female respondents
Age 40-74 years 859
54.8±10.4
2007
Total respondents 2016
Female respondents 1221
Female respondents
Age 40-74 years 864
54.2±10.0
2008
Total respondents 2033
Female respondents 1209
Female respondents
Age 40-74 years 850
53.5±10.0
2009
Total respondents 2985
Female respondents 2169
Female respondents
Age 40-74 years 841
53.7±10.0
2010
Total respondents 3937
Female respondents 2310
Female respondents
Age 40-74 years 1732
53.1±9.3
2011
Total respondents 3975
Female respondents 2333
Female respondents
Age 40-74 years 553
53.8±9.3
2012
Total respondents 4080
Female respondents 2360
Female respondents
Age 40-74 years 1780
53.8±9.2
2013
Total respondents 3722
Female respondents 2167
Female respondents
Age 40-74 years 1773
53.7±8.8
2014
Total respondents 4500
Female respondents 2770
Female respondents
Age 40-74 years 1711
53.8±9.0
2015
Total respondents 4500
Female respondents 2770
Female respondents
Age 40-74 years 1711
54.1±9.0
2016
Total respondents 4500
Female respondents 2767
Female respondents
Age 40-74 years 1747
54.4±9.2
2017
Total respondents 4500
Female respondents 2764
Female respondents
Age 40-74 years 1748
54.4±9.1
2018
Total respondents 4500
Female respondents 2759
Female respondents
Age 40-74 years 1754
54.5±8.7
2019
Total respondents 4500
Female respondents 2756
Female respondents
Age 40-74 years 1795
55.9±9.4
2020
Total respondents 4500
Female respondents 2801
Female respondents
Age 40-74 years 1800
56.3±9.4
KNCSS 2005−2020
Selected 23702 female respondents aged 40-74 as the analysis subjects
Supplementary Materail 1. Selection of analysis subjects flowchart by the year 2005–2020.
