# Supplementary material for: Trends in breast cancer screening rates among Korean women: results from the Korean National Cancer Screening Survey, 2005-2020
Source: Epidemiol Health. 2022 Nov 24;44:e2022111. doi: 10.4178/epih.e2022111 (PMC10396513; doi:10.4178/epih.e2022111)
Supplement: Supplementary Material 2. — Korean National Cancer Screening Survey Questionnaire, 2020. [file epih-44-e2022111-Supplementary-2.docx]

**Supplementary Material 2. Korean National Cancer Screening Survey Questionnaire, 2020.**

**[Interviewer: Answer with the most recent examination.]**

**Q1-1) Have you ever been examined by the following screening methods?**

| **Cancer** | **Methods** | **Response** | |
| --- | --- | --- | --- |
|  |  | ①  Yes | ②  No |
| Stomach | A. Upper GI series |  |  |
|  | B. Upper endoscopy |  |  |
| Liver | C. Liver ultrasonography |  |  |
| Colorectal | D. Stool test (FOBT) |  |  |
|  | E. Colonoscopy |  |  |
| Breast | F. Mammography |  |  |
|  | G. Breast Ultrasonography |  |  |
| Cervical | H. Pap smear |  |  |
|  | I. HPV DNA test |  |  |

**Q1-2) When was the last time you had this screening examination?**

1. **Stomach, Breast, Cervical, Colorectal (Stool test)**

| **Cancer** | **Methods** | **Response** | | |
| --- | --- | --- | --- | --- |
|  |  | ①  Within 1 year (12 months) | ②  Within 2 years (13 – 24 months) | ③  More than 2 years ago (≥ 25 months) |
| Stomach | A. Upper GI series |  |  |  |
|  | B. Upper endoscopy |  |  |  |
| Colorectal | D. Stool test (FOBT) |  |  |  |
| Breast | F. Mammography |  |  |  |
|  | G. Breast Ultrasonography |  |  |  |
| Cervical | H. Pap smear |  |  |  |
|  | I. HPV DNA test |  |  |  |

1. **Liver**

| **Cancer** | **Methods** | **Response** | | |
| --- | --- | --- | --- | --- |
|  |  | ①  Within 6 months | ②  Within 1 year (7 – 12 months) | ③  More than 1 year ago (≥ 13 months) |
| Liver | C. Liver ultrasonography |  |  |  |

1. **Colorectal (Colonoscopy, DCBE)**

| **Cancer** | **Methods** | **Response** | | |
| --- | --- | --- | --- | --- |
|  |  | ①  Within 5 years | ②  Within 10 years | ③  More than 10 years ago |
| Colorectal | E. Colonoscopy |  |  |  |

**Q1-4) How did you pay for the screening examination?**

| **Cancer** | **Methods** | **Response** | | | | | |
| --- | --- | --- | --- | --- | --- | --- | --- |
|  |  | ①  NHI/ NHIC (partially self-paid) | ②  Public health center/ Government (total free of charge) | ③  Private insurance company | ④  Comprehensive medical examination (full amount paid by yourself/ spouse/ children) | ⑤  (your/ spouse’s/ children’s) Workplace checkup | ⑥  Others  (Please write: _______________) |
| Stomach | A. Upper GI series |  |  |  |  |  |  |
|  | B. Upper endoscopy |  |  |  |  |  |  |
| Liver | C. Liver ultrasonography |  |  |  |  |  |  |
| Colorectal | D. Stool test (FOBT) |  |  |  |  |  |  |
|  | E. Colonoscopy |  |  |  |  |  |  |
| Breast | F. Mammography |  |  |  |  |  |  |
|  | G. Breast Ultrasonography |  |  |  |  |  |  |
| Cervical | H. Pap smear |  |  |  |  |  |  |
|  | I. HPV DNA test |  |  |  |  |  |  |

DCBE = double-contrast barium enema; FOBT = fecal occult blood test; GI = gastrointestinal; NHI = National Health Insurance; NHIC = National Health Insurance Corporation; HPV = human papillomavirus.
