# Supplementary material for: Trends in breast cancer screening rates among Korean women: results from the Korean National Cancer Screening Survey, 2005-2020
Source: Epidemiol Health. 2022 Nov 24;44:e2022111. doi: 10.4178/epih.e2022111 (PMC10396513; doi:10.4178/epih.e2022111)
Supplement: Supplementary Material 3. — Distributions (%) of the sociodemographic characteristics of the respondents according to the Korean National Cancer Screening Survey (KNCSS), 2005–2020 [file epih-44-e2022111-Supplementary-3.docx]

Supplementary Material 3. Distributions (%) of the sociodemographic characteristics of the respondents according to the Korean National Cancer Screening Survey (KNCSS), 2005–2020

|  | Survey year | | | | | | | | | | | | | | | | | | | | | | | | | | | | | | | |
| --- | --- | --- | --- | --- | --- | --- | --- | --- | --- | --- | --- | --- | --- | --- | --- | --- | --- | --- | --- | --- | --- | --- | --- | --- | --- | --- | --- | --- | --- | --- | --- | --- |
|  | 2005 | | 2006 | | 2007 | | 2008 | | 2009 | | 2010 | | 2011 | | 2012 | | 2013 | | 2014 | | 2015 | | 2016 | | 2017 | | 2018 | | 2019 | | 2020 | |
| ***Number of respondents* (n)** ^a)^ |  | |  | |  | |  | |  | |  | |  | |  | |  | |  | |  | |  | |  | |  | |  | |  | |
| Total | 970 | | 859 | | 864 | | 850 | | 841 | | 1732 | | 1780 | | 1767 | | 1773 | | 1711 | | 1711 | | 1747 | | 1748 | | 1754 | | 1795 | | 1800 | |
| Underwent screening | 379 | | 348 | | 396 | | 419 | | 464 | | 1058 | | 1077 | | 1254 | | 1058 | | 1133 | | 1049 | | 1100 | | 1112 | | 1106 | | 1119 | | 1143 | |
| ***Mean age* (SD)** |  | |  | |  | |  | |  | |  | |  | |  | |  | |  | |  | |  | |  | |  | |  | |  | |
| Total | 51.7 | (8.8) | 54.8 | (10.4) | 54.2 | (10.0) | 53.5 | (9.6) | 53.7 | (9.6) | 53.1 | (9.3) | 53.8 | (9.3) | 53.8 | (9.2) | 53.7 | (8.8) | 53.8 | (9.0) | 54.1 | (9.0) | 54.4 | (9.2) | 54.4 | (9.1) | 54.5 | (8.7) | 55.9 | (9.4) | 56.3 | (9.4) |
| Underwent screening | 51.7 | (7.7) | 54.1 | (9.5) | 53.9 | (9.2) | 54.4 | (9.2) | 54.1 | (9.1) | 53.1 | (8.9) | 53.8 | (8.8) | 53.6 | (8.8) | 53.1 | (8.6) | 53.6 | (8.7) | 53.9 | (8.6) | 54.9 | (9.2) | 54.2 | (8.9) | 53.8 | (8.3) | 55.3 | (9.1) | 56.0 | (9.0) |
| ***Distributions by subgroup* (%)** ^b)^ |  | |  | |  | |  | |  | |  | |  | |  | |  | |  | |  | |  | |  | |  | |  | |  | |
| Age (yr) ^c)^ |  | |  | |  | |  | |  | |  | |  | |  | |  | |  | |  | |  | |  | |  | |  | |  | |
| 40–49 | 46.9 | | 39.6 | | 39.8 | | 42.1 | | 41.5 | | 39.5 | | 38.6 | | 38.9 | | 37.5 | | 37.1 | | 36.4 | | 35.6 | | 34.9 | | 34.2 | | 30.6 | | 29.8 | |
| 50–59 | 31.5 | | 25.4 | | 25.2 | | 29.3 | | 29.8 | | 31.7 | | 33.5 | | 33.3 | | 34.1 | | 34.2 | | 34.1 | | 34.2 | | 34.2 | | 34.1 | | 31.8 | | 31.2 | |
| 60–69 | 19.1 | | 24.4 | | 29.6 | | 20.9 | | 20.5 | | 20.8 | | 19.7 | | 19.5 | | 20.2 | | 20.2 | | 21.2 | | 22.2 | | 23.0 | | 23.8 | | 23.1 | | 24.3 | |
| 70–74 | 2.5 | | 10.6 | | 5.4 | | 7.8 | | 8.2 | | 8.0 | | 8.2 | | 8.3 | | 8.1 | | 8.5 | | 8.4 | | 8.0 | | 8.0 | | 7.9 | | 14.5 | | 14.7 | |
| Education (yr) |  | |  | |  | |  | |  | |  | |  | |  | |  | |  | |  | |  | |  | |  | |  | |  | |
| ≤11 | 46.2 | | 55.2 | | 49.3 | | 45.8 | | 40.3 | | 30.3 | | 28.8 | | 26.7 | | 17.9 | | 17.3 | | 19.5 | | 16.2 | | 19.4 | | 16.7 | | 19.8 | | 19.0 | |
| 12–15 | 43.7 | | 37.3 | | 42.5 | | 44.6 | | 46.5 | | 54.0 | | 54.7 | | 57.4 | | 59.2 | | 57.7 | | 59.0 | | 56.2 | | 55.3 | | 58.1 | | 57.2 | | 57.3 | |
| ≥16 | 10.2 | | 7.5 | | 8.2 | | 9.6 | | 13.2 | | 15.7 | | 16.5 | | 15.9 | | 22.9 | | 25.0 | | 21.5 | | 27.6 | | 25.3 | | 25.2 | | 23.1 | | 23.7 | |
| Monthly household income ^d)^ |  | |  | |  | |  | |  | |  | |  | |  | |  | |  | |  | |  | |  | |  | |  | |  | |
| Low income | 30.3 | | 33.6 | | 25.7 | | 32.1 | | 30.7 | | 35.1 | | 33.9 | | 29.3 | | 36.5 | | 25.8 | | 25.4 | | 26.2 | | 36.0 | | 25.1 | | 34.4 | | 36.1 | |
| Middle income | 41.9 | | 36.9 | | 38.1 | | 30.1 | | 42.5 | | 34.9 | | 35.5 | | 43.1 | | 31.8 | | 37.1 | | 37.9 | | 43.5 | | 31.1 | | 35.6 | | 35.7 | | 31.8 | |
| High income | 27.8 | | 29.5 | | 36.2 | | 37.8 | | 26.8 | | 30.0 | | 30.6 | | 27.7 | | 31.7 | | 37.0 | | 36.7 | | 30.3 | | 32.9 | | 39.3 | | 29.9 | | 32.2 | |
| Marital status |  | |  | |  | |  | |  | |  | |  | |  | |  | |  | |  | |  | |  | |  | |  | |  | |
| Married | 90.1 | | 84.4 | | 84.9 | | 87.1 | | 88.5 | | 88.1 | | 88.2 | | 93.3 | | 90.4 | | 93.3 | | 93.4 | | 91.1 | | 94.1 | | 93.0 | | 93.2 | | 91.2 | |
| Not married | 0.7 | | 0.7 | | 0.8 | | 0.6 | | 0.9 | | 0.9 | | 1.2 | | 0.5 | | 1.6 | | 1.1 | | 0.7 | | 3.5 | | 1.1 | | 1.6 | | 1.0 | | 0.7 | |
| Others ^e)^ | 9.2 | | 14.9 | | 14.3 | | 12.3 | | 10.6 | | 11.0 | | 10.6 | | 6.2 | | 8.1 | | 5.6 | | 5.9 | | 5.4 | | 4.8 | | 5.4 | | 5.8 | | 8.1 | |
| Residential area |  | |  | |  | |  | |  | |  | |  | |  | |  | |  | |  | |  | |  | |  | |  | |  | |
| Metropolitan | 46.5 | | 46.6 | | 46.8 | | 47.1 | | 46.7 | | 44.0 | | 45.6 | | 44.4 | | 44.6 | | 45.2 | | 46.1 | | 44.6 | | 45.6 | | 44.9 | | 44.4 | | 46.1 | |
| Urban | 39.2 | | 39.8 | | 40.0 | | 42.7 | | 43.1 | | 41.2 | | 40.2 | | 33.3 | | 41.3 | | 47.6 | | 47.7 | | 42.7 | | 44.1 | | 43.3 | | 45.1 | | 44.3 | |
| Rural | 14.3 | | 13.6 | | 13.2 | | 10.2 | | 10.2 | | 14.8 | | 14.2 | | 22.3 | | 14.1 | | 7.2 | | 6.2 | | 12.7 | | 10.3 | | 11.8 | | 10.5 | | 9.7 | |
| Health insurance type |  | |  | |  | |  | |  | |  | |  | |  | |  | |  | |  | |  | |  | |  | |  | |  | |
| National Health Insurance | 94.9 | | 95.2 | | 95.5 | | 95.7 | | 94.8 | | 95.5 | | 95.8 | | 98.2 | | 96.4 | | 97.6 | | 99.3 | | 98.5 | | 99.0 | | 98.4 | | 98.1 | | 99.2 | |
| Medical Aid Program | 5.1 | | 4.8 | | 4.5 | | 4.3 | | 5.2 | | 4.5 | | 4.2 | | 1.8 | | 3.6 | | 2.4 | | 0.7 | | 1.5 | | 1.0 | | 1.6 | | 1.9 | | 0.8 | |

SD = standardized deviation. ^a)^ The crude number of respondents. ^b)^ Some columns do not sum to 100% because of missing data. ^c)^ Respondents were restricted to women 40-74 years of age who had last undergone screening with mammography within a period of 2 years. ^d)^ Low-income, middle-income, and high-income groups were classified according to each year's tertile of household income. ^e)^ Others mean divorced or separated.
