# Supplementary material for: Trends in breast cancer screening rates among Korean women: results from the Korean National Cancer Screening Survey, 2005-2020
Source: Epidemiol Health. 2022 Nov 24;44:e2022111. doi: 10.4178/epih.e2022111 (PMC10396513; doi:10.4178/epih.e2022111)
Supplement: Supplementary Material 4. — Number of respondents underwent organized screening and the organized screening rates (%) by the sociodemographic characteristics according to the Korean National Cancer Screening Survey (KNCSS), 2005–2020 [file epih-44-e2022111-Supplementary-4.docx]

|  | Survey year | | | | | | | | | | | | | | | | | | |  |
| --- | --- | --- | --- | --- | --- | --- | --- | --- | --- | --- | --- | --- | --- | --- | --- | --- | --- | --- | --- | --- |
|  | 2005 | 2006 | 2007 | 2008 | 2009 | 2010 | 2011 | 2012 | 2013 | 2014 | 2015 | 2016 | 2017 | 2018 | 2019 | 2020 | AAPC (95% CI) | | | |
| ***Number of respondents* (n)** ^a)^ |  |  |  |  |  |  |  |  |  |  |  |  |  |  |  |  |  | − |  |  |
| Total respondents | 970 | 859 | 864 | 850 | 841 | 1732 | 1780 | 1767 | 1773 | 1711 | 1711 | 1747 | 1748 | 1754 | 1795 | 1800 |  | − |  |  |
| Underwent organized screening | 222 | 232 | 240 | 300 | 353 | 814 | 825 | 1015 | 817 | 927 | 916 | 919 | 1012 | 953 | 972 | 1050 |  | − |  |  |
| ***Screening rate* (%)** ^b)^ |  |  |  |  |  |  |  |  |  |  |  |  |  |  |  |  |  | − |  |  |
| Total | 22.9 | 27.0 | 27.7 | 35.6 | 41.9 | 47.0 | 46.2 | 57.4 | 46.1 | 54.0 | 53.4 | 52.6 | 57.9 | 54.3 | 54.1 | 58.3 | 6.5* | (3.9 − 9.3) |  |  |
| Age (years) ^c)^ |  |  |  |  |  |  |  |  |  |  |  |  |  |  |  |  |  |  |  |  |
| 40–49 | 18.7 | 23.8 | 22.4 | 26.3 | 35.8 | 41.9 | 39.5 | 54.5 | 46.8 | 52.0 | 54.3 | 45.4 | 59.1 | 55.6 | 55.7 | 56.1 | 7.5* | (5.0 − 10.1) |  |  |
| 50–59 | 30.1 | 33.0 | 32.1 | 40.2 | 45.0 | 53.6 | 53.1 | 64.2 | 47.6 | 59.0 | 55.6 | 53.6 | 57.7 | 57.5 | 56.8 | 60.3 | 5.0* | (2.0 − 8.1) |  |  |
| 60–69 | 22.7 | 30.5 | 32.8 | 43.4 | 53.5 | 48.9 | 48.6 | 53.0 | 46.0 | 53.3 | 51.8 | 61.0 | 58.6 | 54.0 | 55.1 | 62.7 | 6.2* | (3.2 − 9.3) |  |  |
| 70–74 | 12.5 | 16.5 | 19.1 | 47.2 | 32.4 | 40.7 | 43.3 | 54.0 | 37.0 | 45.0 | 45.1 | 57.6 | 51.2 | 36.0 | 43.6 | 51.2 | 11.2 | (- 4.1 − 28.9) |  |  |
| Education (years) |  |  |  |  |  |  |  |  |  |  |  |  |  |  |  |  |  |  |  |  |
| ≤11 | 24.3 | 26.8 | 31.0 | 42.0 | 49.1 | 49.8 | 49.2 | 57.0 | 42.8 | 53.3 | 47.9 | 59.0 | 58.1 | 39.2 | 45.3 | 55.2 | 5.6* | (1.8 − 9.6) |  |  |
| 12–15 | 22.1 | 26.7 | 24.6 | 32.5 | 36.8 | 46.6 | 45.8 | 60.0 | 48.5 | 55.3 | 56.2 | 54.6 | 57.4 | 57.6 | 55.6 | 60.5 | 6.6* | (4.8 − 8.3) |  |  |
| ≥16 | 20.6 | 30.2 | 20.1 | 16.6 | 37.6 | 43.1 | 41.9 | 48.9 | 42.4 | 51.8 | 50.8 | 44.8 | 58.9 | 56.7 | 57.9 | 55.4 | 4.6* | (2.6 − 6.7) |  |  |
| Monthly household income ($) ^d)^ |  |  |  |  |  |  |  |  |  |  |  |  |  |  |  |  |  |  |  |  |
| Low income | 25.5 | 27.2 | 27.0 | 44.9 | 50.8 | 50.7 | 49.7 | 57.2 | 45.3 | 49.0 | 47.1 | 56.5 | 56.4 | 44.3 | 51.1 | 57.0 | 5.6* | (1.5 − 10.0) |  |  |
| Middle income | 20.7 | 29.1 | 31.3 | 30.5 | 38.6 | 45.6 | 44.8 | 59.7 | 44.5 | 56.0 | 54.3 | 51.8 | 54.0 | 55.4 | 52.7 | 59.9 | 5.9* | (3.8 − 8.0) |  |  |
| High income | 23.3 | 24.5 | 24.5 | 32.3 | 37.5 | 44.3 | 43.7 | 54.2 | 48.6 | 55.6 | 56.9 | 50.4 | 63.3 | 59.7 | 59.3 | 58.2 | 7.0* | (4.7 − 9.3) |  |  |
| Residential area |  |  |  |  |  |  |  |  |  |  |  |  |  |  |  |  |  |  |  |  |
| Metropolitan | 21.7 | 26.3 | 25.8 | 33.4 | 40.4 | 49.0 | 47.9 | 53.0 | 50.4 | 58.1 | 53.9 | 51.7 | 58.2 | 57.4 | 58.7 | 56.0 | 7.3* | (5.1 − 9.4) |  |  |
| Urban | 22.1 | 26.6 | 3.1 | 14.2 | 43.1 | 42.1 | 41.1 | 59.6 | 42.6 | 50.9 | 54.7 | 50.8 | 56.5 | 52.9 | 50.7 | 61.1 | 4.8* | (1.7 − 8.0) |  |  |
| Rural | 28.8 | 30.8 | 5.3 | 12.0 | 44.3 | 54.7 | 54.8 | 63.0 | 42.7 | 49.5 | 40.7 | 62.0 | 62.5 | 47.8 | 49.3 | 55.7 | 2.7 | (-1.2 – 6.6) |  |  |

Supplementary Material 4. Number of respondents underwent organized screening and the organized screening rates (%) by the sociodemographic characteristics according to the Korean National Cancer Screening Survey (KNCSS), 2005–2020

AAPC = average annual percent change; CI = confidence interval. ^a)^ The crude number of respondents. ^b)^ The screening rates were calculated by applying survey sample weights. ^c)^ Respondents were restricted to women 40-74 years of age who had last undergone screening with mammography within a period of 2 years. ^d)^ Low-income, middle-income, and high-income groups were classified according to each year's tertile of household income.
