# Supplementary material for: Trends in breast cancer screening rates among Korean women: results from the Korean National Cancer Screening Survey, 2005-2020
Source: Epidemiol Health. 2022 Nov 24;44:e2022111. doi: 10.4178/epih.e2022111 (PMC10396513; doi:10.4178/epih.e2022111)
Supplement: Supplementary Material 5. — Number of respondents underwent opportunistic screening and the opportunistic screening rates (%) by the sociodemographic characteristics according to the Korean National Cancer Screening Survey (KNCSS), 2005–2020 [file epih-44-e2022111-Supplementary-5.docx]

|  | Survey year | | | | | | | | | | | | | | | | | | |  |
| --- | --- | --- | --- | --- | --- | --- | --- | --- | --- | --- | --- | --- | --- | --- | --- | --- | --- | --- | --- | --- |
|  | 2005 | 2006 | 2007 | 2008 | 2009 | 2010 | 2011 | 2012 | 2013 | 2014 | 2015 | 2016 | 2017 | 2018 | 2019 | 2020 | AAPC (95% CI) | | | |
| ***Number of respondents* (n)** ^a)^ |  |  |  |  |  |  |  |  |  |  |  |  |  |  |  |  |  |  |  |  |
| Total respondents | 970 | 859 | 864 | 850 | 841 | 1732 | 1780 | 1767 | 1773 | 1711 | 1711 | 1747 | 1748 | 1754 | 1795 | 1800 |  | − |  |  |
| Underwent opportunistic screening | 157 | 116 | 156 | 119 | 108 | 244 | 252 | 239 | 241 | 206 | 133 | 181 | 100 | 153 | 147 | 93 |  | − |  |  |
| ***Screening rate* (%)** ^b)^ |  |  |  |  |  |  |  |  |  |  |  |  |  |  |  |  |  |  |  |  |
| Total | 16.2 | 13.5 | 18.1 | 14.0 | 12.8 | 14.1 | 14.2 | 13.5 | 13.6 | 12.0 | 7.8 | 10.4 | 5.7 | 8.7 | 8.2 | 5.2 | -6.0* | (-8.2 − -3.9) |  |  |
| Age (years) ^c)^ |  |  |  |  |  |  |  |  |  |  |  |  |  |  |  |  |  |  |  |  |
| 40–49 | 17.1 | 16.8 | 21.6 | 16.0 | 15.2 | 17.4 | 17.9 | 14.6 | 17.0 | 14.2 | 8.0 | 12.9 | 4.8 | 10.3 | 11.1 | 8.0 | -5.3* | (-7.9 − -2.5) |  |  |
| 50–59 | 18.6 | 11.9 | 19.9 | 16.9 | 12.4 | 13.0 | 13.5 | 13.4 | 11.6 | 10.9 | 8.9 | 8.7 | 7.2 | 9.2 | 7.9 | 5.2 | -6.6* | (-8.2 − -4.9) |  |  |
| 60–69 | 11.4 | 13.8 | 13.8 | 8.4 | 12.1 | 10.2 | 9.4 | 15.1 | 10.6 | 10.3 | 5.1 | 8.9 | 5.9 | 7.0 | 6.3 | 3.7 | -6.0* | (-8.9 − -2.9) |  |  |
| 70–74 | 4.2 | 4.4 | 6.4 | 7.4 | 4.5 | 11.6 | 10.8 | 5.4 | 13.9 | 11.0 | 8.7 | 10.3 | 2.9 | 5.0 | 5.8 | 1.9 | -1.7 | (-11.0 − 8.6) |  |  |
| Education (years) |  |  |  |  |  |  |  |  |  |  |  |  |  |  |  |  |  |  |  |  |
| ≤11 | 14.3 | 10.5 | 12.6 | 10.4 | 10.8 | 10.1 | 9.6 | 10.0 | 10.7 | 10.2 | 5.5 | 7.5 | 3.5 | 7.8 | 6.2 | 2.1 | -5.8* | (-8.4 − -3.2) |  |  |
| 12–15 | 16.8 | 15.9 | 22.5 | 16.6 | 13.5 | 14.7 | 15.0 | 15.0 | 13.1 | 10.8 | 6.9 | 8.2 | 5.6 | 7.6 | 7.6 | 4.5 | -8.3* | (-10.6 − -6.0) |  |  |
| ≥16 | 22.7 | 23.8 | 30.4 | 20.3 | 17.4 | 19.7 | 19.5 | 14.3 | 17.2 | 16.1 | 12.1 | 16.5 | 7.7 | 12.0 | 11.4 | 9.4 | -6.3* | (-8.5 − -4.1) |  |  |
| Monthly household income ($) ^d)^ |  |  |  |  |  |  |  |  |  |  |  |  |  |  |  |  |  |  |  |  |
| Low income | 14.6 | 8.6 | 9.6 | 8.5 | 8.2 | 9.8 | 9.6 | 10.3 | 12.5 | 12.7 | 6.6 | 7.2 | 4.4 | 6.4 | 5.3 | 3.7 | -6.5* | (-11.0 − -1.7) |  |  |
| Middle income | 14.8 | 13.1 | 15.7 | 12.3 | 13.6 | 13.5 | 13.5 | 13.3 | 11.9 | 9.7 | 5.1 | 9.0 | 4.4 | 8.6 | 7.8 | 6.3 | -5.9* | (-8.3 − -3.4) |  |  |
| High income | 20.0 | 18.4 | 26.2 | 20.6 | 17.4 | 19.7 | 20.2 | 17.1 | 16.6 | 13.9 | 11.4 | 15.1 | 8.4 | 10.3 | 11.9 | 5.7 | -6.6* | (-8.8 − -4.3) |  |  |
| Residential area |  |  |  |  |  |  |  |  |  |  |  |  |  |  |  |  |  |  |  |  |
| Metropolitan | 16.2 | 13.3 | 17.2 | 14.8 | 13.7 | 11.7 | 12.0 | 14.7 | 13.7 | 10.6 | 6.2 | 11.9 | 6.7 | 8.1 | 6.9 | 5.6 | -6.5* | (-9.0 − -3.9) |  |  |
| Urban | 17.1 | 13.5 | 18.6 | 14.6 | 11.3 | 15.0 | 14.8 | 14.0 | 13.4 | 13.9 | 9.1 | 10.2 | 5.1 | 9.1 | 10.3 | 4.9 | -5.1* | (-7.7 − -2.5) |  |  |
| Rural | 13.7 | 14.5 | 19.5 | 7.9 | 15.1 | 15.1 | 15.1 | 10.6 | 14.1 | 8.8 | 9.4 | 5.8 | 4.4 | 9.7 | 4.7 | 4.6 | -7.2* | (-10.5 − -3.7) |  |  |

Supplementary Material 5. Number of respondents underwent opportunistic screening and the opportunistic screening rates (%) by the sociodemographic characteristics according to the Korean National Cancer Screening Survey (KNCSS), 2005–2020

AAPC = average annual percent change; CI = confidence interval. ^a)^ The crude number of respondents. ^b)^ The screening rates were calculated by applying survey sample weights. ^c)^ Respondents were restricted to women 40-74 years of age who had last undergone screening with mammography within a period of 2 years. ^d)^ Low-income, middle-income, and high-income groups were classified according to each year's tertile of household income.
