# Supplementary material for: Trends in breast cancer screening rates among Korean women: results from the Korean National Cancer Screening Survey, 2005-2020
Source: Epidemiol Health. 2022 Nov 24;44:e2022111. doi: 10.4178/epih.e2022111 (PMC10396513; doi:10.4178/epih.e2022111)
Supplement: Supplementary Material 6. — Trends in breast cancer screening rates with recommendation by the payment method, 2005–2020. The marker (×) denotes an observed screening rate. The solid line denotes a significantly increasing or decreasing trend; the densely dotted line denotes a non-significant change. * p-value for the trend in annual percent changes (APCs) <0.05. (A) The pink and green lines denote the trends in women who underwent organized screening and women who underwent opportunistic screening, respectively. [file epih-44-e2022111-Supplementary-6.pptx]

## Slide 1
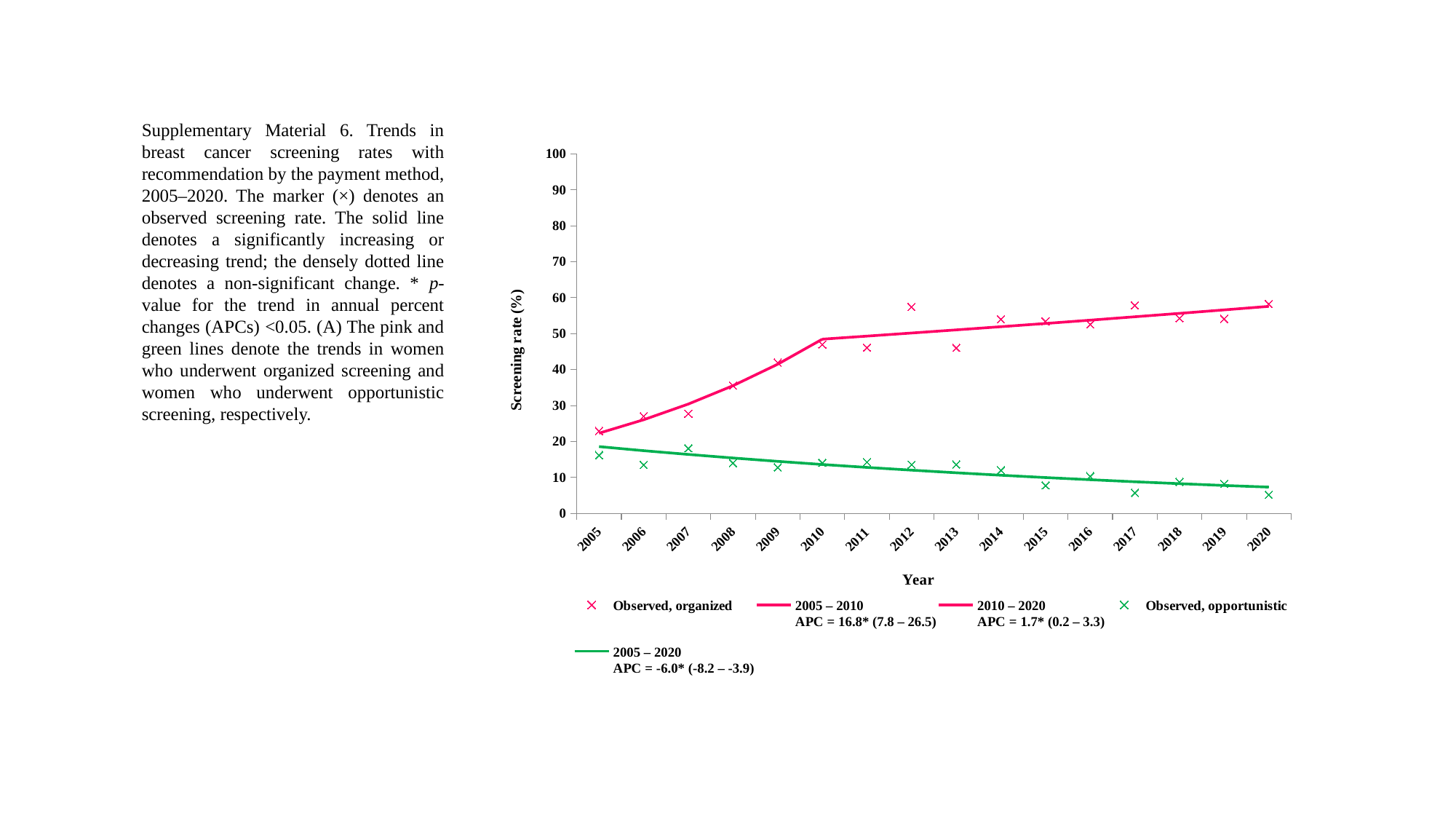

Supplementary Material 6. Trends in breast cancer screening rates with recommendation by the payment method, 2005–2020. The marker (×) denotes an observed screening rate. The solid line denotes a significantly increasing or decreasing trend; the densely dotted line denotes a non-significant change. * p-value for the trend in annual percent changes (APCs) <0.05. (A) The pink and green lines denote the trends in women who underwent organized screening and women who underwent opportunistic screening, respectively.
### Chart
| Category | Observed, organized | 2005 – 2010
APC = 16.8* (7.8 – 26.5) | 2010 – 2020
APC = 1.7* (0.2 – 3.3) | Observed, opportunistic | 2005 – 2020
APC = -6.0* (-8.2 – -3.9) |
|---|---|---|---|---|---|
| 2005 | 22.89 | 22.29 | None | 16.19 | 18.56 |
| 2006 | 27.01 | 26.04 | None | 13.5 | 17.43 |
| 2007 | 27.71 | 30.42 | None | 18.09 | 16.38 |
| 2008 | 35.55 | 35.53 | None | 14.02 | 15.39 |
| 2009 | 41.92 | 41.5 | None | 12.81 | 14.46 |
| 2010 | 46.99 | 48.47 | 48.47 | 14.06 | 13.59 |
| 2011 | 46.15 | None | 49.32 | 14.23 | 12.77 |
| 2012 | 57.44 | None | 50.18 | 13.55 | 12.0 |
| 2013 | 46.08 | None | 51.05 | 13.6 | 11.27 |
| 2014 | 54.04 | None | 51.94 | 11.98 | 10.59 |
| 2015 | 53.43 | None | 52.84 | 7.77 | 9.95 |
| 2016 | 52.62 | None | 53.76 | 10.32 | 9.35 |
| 2017 | 57.9 | None | 54.7 | 5.72 | 8.78 |
| 2018 | 54.32 | None | 55.65 | 8.73 | 8.25 |
| 2019 | 54.13 | None | 56.62 | 8.2 | 7.75 |
| 2020 | 58.28 | None | 57.6 | 5.16 | 7.29 |
